# Biopolymer Nanoparticles for Nose-to-Brain Drug Delivery: A New Promising Approach for the Treatment of Neurological Diseases

**DOI:** 10.3390/jfb13030125

**Published:** 2022-08-24

**Authors:** Nicolò Montegiove, Eleonora Calzoni, Carla Emiliani, Alessio Cesaretti

**Affiliations:** 1 Department of Chemistry, Biology and Biotechnology, Biochemistry and Molecular Biology Section, University of Perugia, Via del Giochetto, 06123 Perugia, Italy; 2 Centro di Eccellenza sui Materiali Innovativi Nanostrutturati (CEMIN), University of Perugia, Via del Giochetto, 06123 Perugia, Italy

**Keywords:** biopolymers, nanoparticles, blood–brain barrier, drug delivery systems, nose-to-brain administration, neurodegenerative disorders, glioblastoma, lysosomal storage diseases

## Abstract

Diseases affecting the central nervous system (CNS) are among the most disabling and the most difficult to cure due to the presence of the blood–brain barrier (BBB) which represents an impediment from a therapeutic and diagnostic point of view as it limits the entry of most drugs. The use of biocompatible polymer nanoparticles (NPs) as vehicles for targeted drug delivery to the brain arouses increasing interest. However, the route of administration of these vectors remains critical as the drug must be delivered without being degraded to achieve a therapeutic effect. An innovative approach for the administration of drugs to the brain using polymeric carriers is represented by the nose-to-brain (NtB) route which involves the administration of the therapeutic molecule through the neuro-olfactory epithelium of the nasal mucosa. Nasal administration is a non-invasive approach that allows the rapid transport of the drug directly to the brain and minimizes its systemic exposure. To date, many studies involve the use of polymer NPs for the NtB transport of drugs to the brain for the treatment of a whole series of disabling neurological diseases for which, as of today, there is no cure. In this review, various types of biodegradable polymer NPs for drug delivery to the brain through the NtB route are discussed and particular attention is devoted to the treatment of neurological diseases such as Glioblastoma and neurodegenerative diseases.

## 1. Introduction

Pharmaceutical research aims at identifying new strategies to modify the course of diseases and improve the patient’s quality of life. This is certainly an important challenge, especially for therapeutic applications that involve the administration of the drug in specific anatomical sites, such as the central nervous system (CNS). In this region, the presence of the blood–brain barrier (BBB), which protects the brain parenchyma from the bloodstream, constitutes a major obstacle that impedes therapeutics to carry out their pharmacological action in situ [1,2]. The most immediate strategy to reach the brain is to physically overcome the BBB through direct intrathecal administration. However, this type of method is certainly invasive, painful, and may produce some unfortunate side effects. The use of biocompatible polymer nanoparticles (NPs) as drug delivery systems is an alternative approach that is gaining growing interest among the possible strategies for the targeted transport of drugs and molecules to the brain for the treatment of all those degenerative diseases involving the CNS [3,4,5]. NPs generally have a diameter between 1 and a few hundred nanometers but NPs with radii of about 100 nm are those with the best pharmacokinetic properties [3,6]. NPs can convey the therapeutic molecule internally (nanocapsules) or by adsorption on their surface (nanospheres). The advantages deriving from the use of nanoparticulate systems concern the possibility of obtaining a controlled and prolonged release of the drug and modifying its distribution and consequent clearance, in order to have, on the one hand, an increase in therapeutic efficacy and on the other hand, a reduction in side effects [3,5]. Moreover, the use of biopolymer-based NPs guarantees, in addition to the specific targeting of the drug or molecule of interest, extremely important peculiarities such as the biocompatibility and biodegradability of the system [3,7,8,9]. The use of nanocarriers has a very high potential for the administration of therapeutic molecules to the brain as, by virtue of their size, are prone to overcoming anatomical barriers such as BBB. However, the route of administration of these carriers remains a crucial point as it is necessary to be able to provide a suitable amount of drug without it being eliminated. A compelling strategy to bypass the obstacle constituted by the BBB and reach the CNS with a satisfying dose of the therapeutic molecule is represented by the nasal administration route defined as nose-to-brain (NtB) drug delivery. The nasal cavity, lined by the nasal mucosa, represents by its anatomy the only contact region between the CNS and the external environment, and therefore the most direct and non-invasive way of accessing the brain [10,11,12]. The nasal route is conventionally used for the administration of drugs for the treatment of local diseases such as rhinitis, nasal infections, and allergic phenomena. In recent years, however, this administration route has aroused growing interest as it has been exploited for the systemic delivery of various drugs, as well as nucleic acids, peptides, proteins, and vaccines [13]. Furthermore, abundant vascularization increases the drug absorption rate and, consequently, allows a rapid onset of the therapeutic effect. It is an easy access route compared to other mucous membranes in the body, non-invasive, and essentially painless. Further to this, the strategy of loading drugs into carriers and delivering them through the nose could potentially increase their access to the CNS. Therefore, NPs may represent an advantage in the administration of drugs to the CNS through the NtB route since they are able to protect the drug from degradation and increase its bioavailability. By doing so, drugs can indeed reach the brain in sufficient quantities, making this route an outstanding approach for the prompt delivery of CNS-active molecules. In this review, biopolymer-based NP drug delivery systems administered through the NtB route will be investigated together with their most recent applications in the treatment of the pathologies involving the CNS.

## 2. Blood-Brain Barrier (BBB)

The blood–brain barrier (BBB) is the natural interface between the brain and the rest of the body. Its function is to protect the brain tissue and to regulate the exchanges with blood circulation. It is formed by the endothelial cells that line the walls of the capillary vessels, by the glial cells, which have a nutritional and supportive function for the nervous system, and by the basement membrane and pericytes that create a supporting system [2,14]. Compared to the peripheral vessels, those of the BBB have a peculiarity: their endothelial cells are joined by tight junctions (TJs). TJs form a compact and particularly selective structure, which allows the passage of essential nutrients and oxygen, but blocks all hydrophilic or large molecules. The presence of BBB is essential, as it protects the brain from either infections or chemicals circulating in the blood [14,15]. On the other hand, however, it also hinders the passage of therapeutic molecules that might be needed in the brain. It is estimated that the BBB excludes access to the brain of 98% of small molecules and the totality of large molecules endowed with therapeutic action [16,17]. The BBB is highly selective allowing the passage through simple diffusion of only certain molecules such as water, carbon dioxide, and oxygen; glucose is capable of passing through this membrane by employing a facilitated diffusion channel, but other molecules of similar size are not able to cross the BBB. Many of the CNS-active drugs, such as peptide and protein drugs, are too large and hydrophilic to pass the BBB from the systemic circulation, and furthermore, when administered orally, are rapidly degraded by gastrointestinal enzymes. High lipophilicity is the main characteristic of the BBB; conversely, most exogenous molecules exhibit significant hydrophilicity, which prevents them from simply crossing it by diffusion. In this category the nutrients necessary to ensure the correct functioning of the CNS are also included, e.g., amino acids, nucleotides, low-molecular-weight peptides, and above all D-glucose, which is the main source of energy for the brain. In fact, these molecules do not cross the endothelium by simple diffusion but need to make use of specific membrane transporters.

### 2.1. Passive and Active Diffusion across the BBB

**Figure 1:**
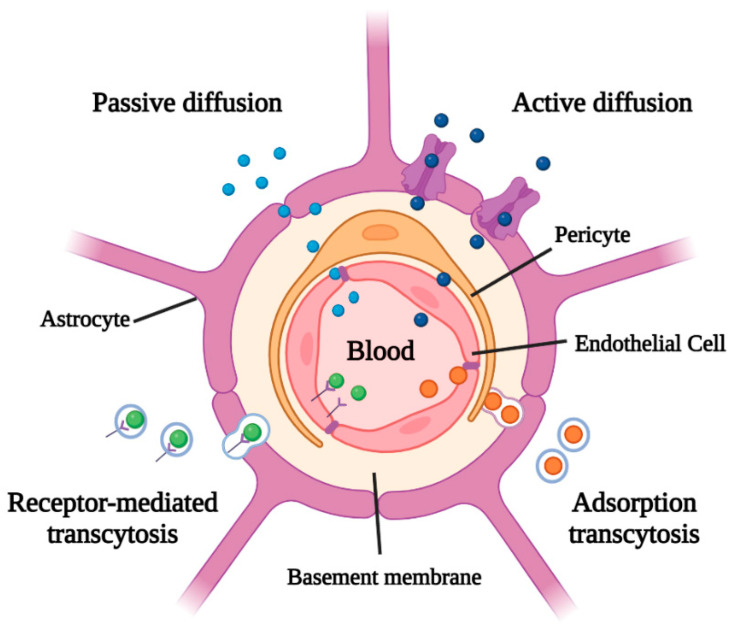
Molecule transport mechanisms through the blood–brain barrier (BBB).

The majority of the drugs have hydrophilic characteristics that prevent their crossing of the BBB, making it necessary to develop new strategies to deliver drugs to the CNS. The transport of substances through the BBB can occur through various mechanisms (Figure 1 and Table 1); in the case of small lipophilic molecules, a passive diffusion process through the endothelial cells is operative [1]. Other nutrients, on the other hand, follow specific transcellular transport routes that ensure that the brain receives all the nutrients it needs. This is the case of glucose, whose transport through the BBB is ensured by the presence of the specific transporter GLUT-1; similarly, essential amino acids are also supplied to the CNS by the LAT1 transporter [1,18,19]. The BBB is also characterized by active diffusion phenomena thanks to the presence of transport pumps belonging to the family of the ATP-binding cassette (ABC) transporters, i.e., integral membrane proteins that use the energy deriving from the hydrolysis of ATP to expel solutes across the cell membrane [20,21]. P-glycoprotein (P-gp) is currently the most studied efflux pump and is responsible for multidrug resistance type 1 (MDR1) [1,22]. In fact, these transmembrane pumps arise as an important protection mechanism against xenobiotic agents potentially toxic but contribute to resistance to anticancer agents in both tumor and normal tissues. Inhibitors of this pump, both natural (i.e., anionic gums and alginates) and synthetic (i.e., polyethylene glycols [PEG], poloxamers such as Pluronic^®^ P85, dendrimers, and thiomers) have been studied. Several studies are now underway aimed at inhibiting this efflux pump to enhance brain targeting delivery [23]. Fernandes et al. demonstrated that PEGylated PLGA NPs (obtained by nanoprecipitation method and with an average diameter of about 100 nm) used for the transport of coumarin C75 for the treatment of Parkinson’s disease, were able to inhibit the effect of P-gp efflux pump in hCMEC/D3 cells favoring the release of the therapeutic molecule [24].

### 2.2. Transcytosis across the BBB

For large molecules, such as proteins, lipoproteins, and peptides larger than 10 amino acids, transcytosis processes allow these molecules to reach the CNS. Roughly speaking, two processes are observed: adsorption transcytosis and receptor-mediated transcytosis (Figure 1 and Table 1) [1,27,28]. The former is a slow and non-selective process in which the endothelial cells incorporate by endocytosis those macromolecules linked in a non-specific way to the membrane surface; an example is represented by positively charged proteins that accumulate on the surface through electrostatic interactions with the negative charges present on the endothelium membrane [26,29]. Receptor-mediated transcytosis, on the other hand, is a slow but selective process in which endothelial cells incorporate those macromolecules that interact specifically with a receptor present on the surface of the membrane; then these substances spread through the endothelium and exits by exocytosis on the opposite side. One of the best-known processes of receptor-mediated transcytosis is clathrin-mediated endocytosis which allows the transport of molecules such as lipoproteins, insulin, and transferrin [30,31]. Other ligands can be used to target the different receptors of the BBB such as folate receptor (FR), lactoferrin receptor (LfR), low-density lipoprotein receptor (LDLR), low-density lipoprotein receptor-related protein (LRP), and transferrin receptor (TfR) [32].

## 3. Biopolymer Nanoparticles (NPs) in the Treatment of CNS Diseases

**Figure 2:**
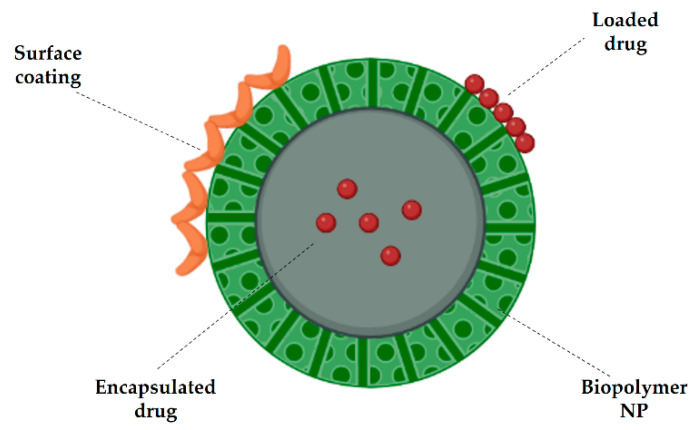
Biopolymer basic structure of a nanoparticle (NP) with encapsulated drug (nanocapsule) or loaded drug (nanosphere). Surface coating can also be present to promote NP penetration.

The development of drugs for the treatment of CNS diseases, including some types of cancer, dementia, neurodegenerative diseases (e.g., Alzheimer’s and Parkinson’s diseases), and lysosomal storage diseases (LSDs) affecting the brain, requires considerable effort inasmuch as it has to deal with the presence of the BBB which represents a major obstacle for the targeted administration of drugs. Among the various strategies currently used to release drugs into the CNS, such as temporary disruption of the TJs in the BBB, chemical modification of drugs, and direct delivery into the brain by surgery, the use of NPs is one of the most interesting techniques [33,34,35,36,37]. NPs represent ideal carriers for drugs that can be either encapsulated inside the particle or simply loaded on its surface by absorption or chemical binding with the polymer itself [38,39] (Figure 2). The use of biodegradable NPs as molecule transporters is one of the most promising strategies for developing controlled-release systems (CRSs). The fundamental requirement for a biomaterial to be used in this sense is its biocompatibility, that is the ability to be metabolized without any harmful effects. In fact, a biodegradable polymer is a polymer that undergoes processes of degradation in vivo. Under certain specific conditions, these biopolymers can spontaneously arrange in self-assemblies of nanometric dimensions (ranging from 1 to 1000 nm) which grant them the name of nano-biopolymers [40]. Biopolymer NPs have been widely used as vehicles for drugs as they provide a series of advantages ranging from the administration of non-water-soluble drugs to the protection of unstable compounds against degradation [3,41,42,43]. The use of NPs is useful for delivering not only drugs but also nucleic acids and therapeutic proteins [33,44,45,46,47,48,49]. The biopolymer NPs used for drug delivery in the CNS can be obtained from natural polymers such as chitosan, sodium alginate, and gelatin; or from synthetic polymers such as polylactic acid (PLA), polyglycolic acid (PGA), poly lactic-co-glycolic acid (PLGA), polybutyl cyanoacrylates (PBCA), and polycaprolactone (PCL) [3,11,49,50,51,52,53]. Despite being synthetic polymers, they can be broken down into oligomers and monomers which are further eliminated through normal metabolic pathways, such as the Krebs cycle [3,54,55]. In addition, these types of systems need to possess other essential properties and fulfill specific tasks: (i) the ability to cross the body’s anatomical barriers, typically the BBB or the ophthalmic barrier, (ii) the possibility of controlling the concentration of the drug over time, and (iii) the capability of releasing active molecules at the site of action. By virtue of their nanometric size and the possibility of being specifically tailored for targeted delivery and controlled release, biopolymer NPs represent the flagship among drug-delivery systems. In fact, they can be administered in various ways and in different regions of the body allowing for their access to target cells and tissues.

### Biopolymer NP Penetration Mechanisms

**Figure 3:**
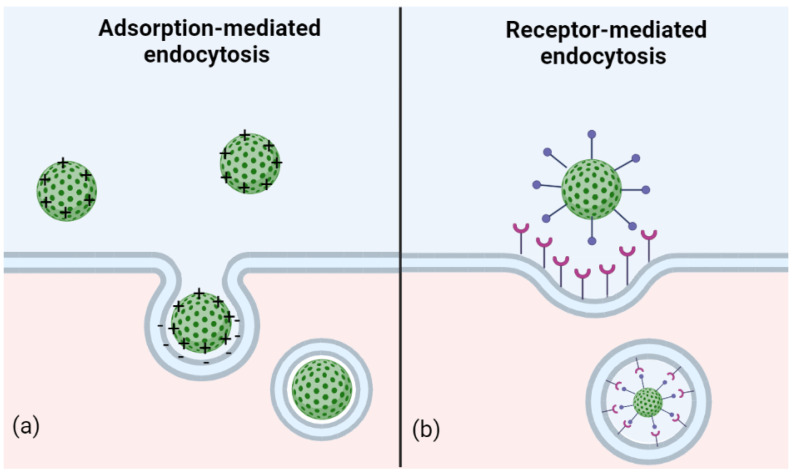
Active transport of nanoparticles (NPs): (**a**) adsorption-mediated endocytosis takes place by electrostatic interactions between the surfaces of NPs and the endothelial membrane and (**b**) receptor-mediated endocytosis originates from the recognition of a ligand on the NP by a membrane receptor of endothelial cell. In both cases, membrane invagination occurs and results in the internalization of the NPs into transport vesicles.

As is the case with other molecules, the penetration of NPs through the BBB can be described by two main mechanisms: passive transport, i.e., simple diffusion; and active transport, which involves energy consumption in the form of ATP [33,56,57]. Just like small lipophilic molecules able to cross the BBB by means of passive diffusion through the endothelial cells, NPs can exploit this function as well, owing to their small size. Moreover, their ability to passively permeate the membrane can be increased by adding cationic charges and lipid molecules to the NP surface [33,56,58]. Furthermore, a PEG-coating is often added to cationic NPs to improve their blood circulation time, avoid the absorption of proteins, escape the immune system, inhibit hemolysis or aggregation of erythrocytes, provide colloidal stability, and protect the carried therapeutic molecule from enzymatic degradation [59]. The main active transport mechanisms, on the other hand, are represented by adsorption-mediated endocytosis and receptor-mediated endocytosis (Figure 3 and Table 2) [33,60,61,62].

In adsorption-mediated endocytosis, electrostatic interactions take place between the positively charged NPs and the negatively charged microdomains present on the cytoplasmic membrane. In this case, the NPs undergo surface modifications by binding with molecules such as albumin and the transactivating transcriptional activator peptide (TAT peptide), which allow electrostatic interactions with the cell membrane [63,64,65,66,67]. Receptor-mediated endocytosis is probably the most commonly used transport mechanism for the administration of NPs as it exploits the receptors expressed on the apical surface of BBB endothelial cells. In particular, the NPs are modified to bring on their surface antigens that can bind to specific receptors; as a result of the bond formation, a vesicle is arranged by the invagination of the membrane, which will allow the NPs to be conveyed inside the cell [64,68].

**Table 2:** Ligands used to coat NPs and promote CNS penetration.

Ligand	Penetration Mechanism	Ref.
Albumin	Adsorption-mediated endocytosis	[63,69]
TAT peptide	Adsorption-mediated endocytosis	[63,64,65,66,67]
Insulin	Receptor-mediated endocytosis	[12,63,70,71]
ApoE	Receptor-mediated endocytosis	[63,72,73]
Transferrin	Receptor-mediated endocytosis	[63,74]
LDL	Receptor-mediated endocytosis	[63,75]
Glutathione	Receptor-mediated endocytosis	[63,76,77]
OX26	Receptor-mediated endocytosis	[63,78,79]

Polymer NPs that exploit these penetration routes have been used for the treatment of a broad spectrum of pathologies involving the CNS, such as Glioblastoma (GBM) or neurodegenerative diseases. To give an example, Sawyer et al. demonstrated that rats affected by brain tumors and treated with camptothecin-loaded PLA NPs prepared using the single-emulsion method and with an average diameter of ~100 nm, showed much higher median survival than the same models treated with either unloaded NPs or free camptothecin [80]. Moreover, in the case of neurodegenerative diseases, there are many examples of polymeric formulations used to deliver therapeutic molecules both in vitro and in animal models. For instance, PEG-co-poly (ε-caprolactone) (PEG-PCL) has been exploited to encapsulate ginkgolide B (GB), a neuroactive ingredient that is commonly used to treat Parkinson’s disease (PD). PEG-PCL-GB NPs prepared via an antisolvent precipitation and with a diameter of ~90 nm, were administered orally to rats, where the drug exhibited significantly higher pharmacokinetics than in its free form [81]. In fact, PEG is the most widely used ligand to date as it provides the stability of NPs in biological media such as blood, it is also demonstrated that a PEG coating induces a stealth effect on NPs helping them to escape the attack of the immune system such as the macrophage action [82]. Similarly, Mittal et al. administered estradiol-loaded PLGA NPs to rat models of Alzheimer’s disease (AD), mimicking a post-menopausal condition, since low estradiol levels are considered a risk factor for developing post-menopausal AD. Estradiol-loaded PLGA NPs with ~130 nm of diameter and prepared using the single-emulsion method resulted effective in preventing the expression of amyloid-beta 42 in the rat model of the disease [83].

## 4. Nose-to-Brain (NtB) Drug Delivery

As it has been clearly stated before, the BBB, which shields the brain parenchyma from the bloodstream, represents a considerable impediment to the in situ pharmacological action of currently available therapeutics. The main problem of treating some pathologies affecting the CNS is thus represented by the difficulty of the therapeutics of crossing the BBB, inasmuch as macromolecular drugs, such as peptide and protein drugs, are too large and too hydrophilic to penetrate the BBB from systemic circulation. Moreover, when administered orally, therapeutic agents are rapidly degraded by the first-pass effect of gastrointestinal and hepatic enzymes such as cytochromes P450 (CYPs) and uridine 5′-diphospho-glucuronosyltransferase (UGT) [84]. A potential and promising strategy to bypass the obstacle represented by the BBB is the transport of drugs and therapeutic peptides and proteins through the nose-to-brain (NtB) route [11,13,85,86,87,88]. This pathway involves the olfactory and trigeminal nerve systems that originate in the brain and end in the nasal cavity, namely in the olfactory neuroepithelium and the respiratory epithelium, respectively [11,85,89,90]. The olfactory region represents the only portion in which the CNS is directly in contact with the external environment, and therefore the most direct and non-invasive way of accessing the brain. Drugs are typically administered via the nasal route to treat harmless local disorders such as rhinitis and nose infections. In recent years, however, this route of administration has aroused growing interest, as it has been exploited for the systemic delivery of various drugs, as a viable alternative to the oral and parenteral route, avoiding gastrointestinal degradation and the hepatic effect typical of oral administration [11,91]. The rich vascularization of the nasal mucosa also increases the absorption rate of the drug and, consequently, allows a rapid onset of the therapeutic effect. It is an easy access route compared to other mucous membranes in the body and it is not invasive and essentially painless [13,90,92,93]. The fact that the olfactory region could represent a portal for the entry of molecules and agents directly into the brain was demonstrated for the first time by inoculating the vesicular stomatitis virus into the nasal epithelium of mice, which reached the CNS via the olfactory nerve [94].

### 4.1. Anatomy of the Nasal Cavity and Drug Delivery Pathways

**Figure 4:**
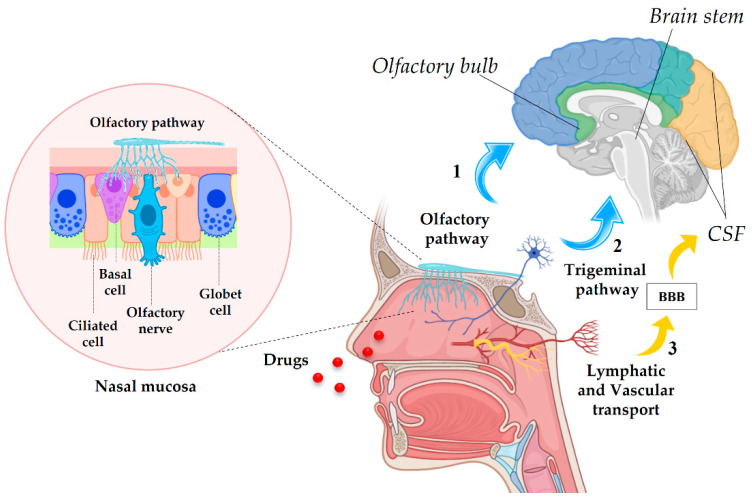
Nose-to-brain (NtB) drug delivery pathways: (1) intracellular pathway from olfactory nerve to the olfactory bulb with a focus on the nasal mucosa, (2) intracellular pathway from trigeminal nerve to the brain stem, and (3) lymphatic and vascular system route to cerebrospinal fluid (CSF) across the BBB.

The nasal cavity is made up of three regions: the vestibular, the respiratory and the olfactory regions. The vestibular region is the outermost one, represents the front portion of the nasal cavity, and is rich in mucus and hair cells which have the function of protecting this anatomical area from potentially harmful external agents. Above the vestibular region, the respiratory region, which is the largest of all areas of the nasal cavity, can be found. This is highly vascularized and includes the trigeminal nerve. The vessels allow the passage of small molecules into the systemic circulation, while the trigeminal neurons allow the transport of molecules and drugs into the brain. The highest region of the nasal cavity is called the olfactory region, which is also somewhat vascularized and hosts the olfactory nerve [13,95,96]. When the drugs are introduced into the nasal cavity via the vestibular region, those molecules that are not retained by mucus and hair cells further proceed to the respiratory and olfactory regions. From here, the transport of drugs to the brain can follow different pathways, namely: (1) transport mediated by the trigeminal nerve, (2) transport mediated by the olfactory nerve, and (3) lymphatic and vascular transport (Figure 4). Among these, the trigeminal and olfactory pathways are certainly the best-known and most studied mechanisms for the transport of NtB drugs [13,92].

The transport of molecules to the brain through the trigeminal and olfactory nerve routes involves a relatively slow intraneuronal pathway in which the drug moves from the olfactory and respiratory epithelium to the olfactory sensory neurons and peripheral trigeminal neurons, respectively, via the process of endocytosis. In this way, the drug is transported to different parts of the CNS; in particular, the intracellular pathway supplies the drug to the olfactory bulb by the olfactory nerve, and to the brain stem by the trigeminal nerve [92,97,98]. The lymphatic system of the nasal cavity and the adjacent vessels are another way of transporting substances to the brain, as they are directly in contact with the cerebrospinal fluid (CSF) [99]. Another transport mechanism is transcellular transport responsible for the release of mainly lipophilic molecules through a process of passive or receptor-mediated diffusion, together with the paracellular pathway in which the transport of molecules to the olfactory lobe and brain stem takes place respectively from the olfactory epithelium and the respiratory epithelium. The physicochemical properties, the nature of the molecules, and the physiological conditions may determine one way of transport rather than another [11,13]. If on the one hand, the NtB route of administration has a high potential for the treatment of all those pathologies involving the CNS, on the other hand, it still has limitations due to the low dose of drugs that can reach the brain as a consequence of the low permeability of molecules through the mucosa, mucociliary clearance, and enzymatic degradation. In light of the above, it is clear that in situations where brain targeting is essential, it is necessary to develop drug delivery systems capable of improving the absorption of the drug from the olfactory and respiratory regions of the nasal cavity to the brain [13].

### 4.2. NPs through the NtB Route

NPs represent a powerful carrier for the administration of drugs, peptides, proteins, and nucleic acids to the CNS through the NtB route and implement the enormous potential of this novel approach, since they can protect the drug from biological and chemical degradation, thus increasing its bioavailability [11,100]. Furthermore, NPs are able to increase the therapeutic effect of the transported drug and the uptake by the brain, also decreasing the side effects of typical drug administration in this region [101,102,103]. In order to improve stability and transmembrane penetration, and to increase the residence time of the formulation in the nasal cavity, these systems may include, in addition to the drug, enzymatic inhibitors, absorption promoters, and mucoadhesive polymers [104,105].

Among nanocarriers, biopolymer NPs are probably the most studied systems due to their biocompatibility and degradability and, as of today, their possible application is being investigated for the treatment of diseases that affect the CNS. The targeted achievement of the brain through the NtB route resorting to this type of carrier involves both the use of natural and synthetic polymers. Chitosan, for example, was among the first polymers to be studied in this sense because, in addition to the inherent characteristics of biodegradability and biocompatibility, it also features bioadhesive properties by having a net positive charge, increases permeability through the mucosa, and reduces mucociliary clearance [11,106,107,108,109]. Chitosan NPs generally have a diameter of around 200 nm and if coated with antibodies, they are perfectly capable of overcoming the BBB, protecting the drug transported from degradation [106]. There are many studies conducted with chitosan NPs administered through the NtB route. Feng et al., for example, have created a nasal spray based on chitosan NPs loaded with the basic fibroblast growth factor (bFGF), an important neurotransmitter able to promote the proliferation of neuronal precursors and therefore an excellent candidate for the treatment of neurodegenerative diseases. It has been shown that the administration bFGF NPs in the nasal cavity of Sprague-Dawley rats allowed an increase in bFGF levels in the brain compared to the administration of the uncomplexed molecule [110,111]. Other studies have been conducted to evaluate the efficacy of NPs of galantamine/chitosan and piperine/chitosan complexes for the AD treatment. In particular, the administration of chitosan/galantamine complex NPs induced a significant decrease in the levels of malondialdehyde (MDA) and tumor necrosis factor-alpha (TNF-α) in the treated groups compared to the controls. Similarly, the piperine/chitosan complex NPs induced an improvement in the cognitive conditions of the treated rats, together with an evident inhibition of the acetylcholine esterase activity and antioxidant effect. In both cases, the chitosan/drug complex was produced by the gelation method to obtain NPs with an average size of ~200 nm [110,112,113]. PLGA is another highly studied biopolymer for this type of drug delivery. Besides the characteristics of biocompatibility and biodegradability, it is able to increase the stability of the transported payload and allow the encapsulation of the drugs to be later released. Compared to natural polymers such as chitosan, PLGA has a lower mucoadhesive capacity; however, it is possible to cover the NPs with mucoadhesive substances such as chitosan itself or PEG to improve retention in the nasal cavity [114,115,116]. PLA is also a widely used biopolymer for the synthesis of NPs to be administered through the NtB drug delivery mechanism. As is the case with other biopolymers, it is possible to exploit the characteristics of biocompatibility and biodegradability of PLA for long-term drug administration, also by virtue of its reduced immunogenicity [48,49,117]. Recently, the study of NPs based on polyethylenimine (PEI), a polycationic light-weight polymer that is exploited for the transport of proteins and nucleic acids, is also enjoying great success. In fact, PEI has a higher charge/mass ratio than other cation polymers, and this allows it to bind to the C-terminal groups of proteins or peptides, protecting them from the action of proteases. It is therefore very interesting as it allows large proteins to be conveyed from the nose directly to the brain [118]. Albeit it has been shown that PEI can be significantly cytotoxic, this cytotoxicity decreases as the size of the NPs decreases, making this polymer potentially usable for this type of drug delivery system [119].

In general, the administration of drug–NP complexes for the treatment of pathologies affecting the CNS via the NtB route must however take into account a series of factors that can influence this mechanism of administration [111]. One of all is the size of the NPs which must have a dimension that allows migration through the mucous membranes to the CNS; in fact, too large particles could be retained. It has been reported that NPs with an average size up to 200 nm could be efficiently transported transcellularly via the intranasal route [120]. The group of Mistry et al. investigated the NtB administration of chitosan-coated polystyrene (C-PS) and polysorbate-coated polystyrene (P80-PS) NPs, with sizes ranging from 100 to 200 nm, in mice. It was shown that for both types of NPs, the size found in the olfactory cells was up to 100 nm, which was therefore assumed to be the maximum usable diameter. Moreover, no NPs were found in olfactory bulbs, suggesting that only NPs with a diameter of less than 100 nm could be transported via the olfactory axons to the brain [100,121]. Another factor influencing this route of administration is the modification of the surface of the NPs to favor migration through the olfactory route. Surface modification with PEG is one of the most viable strategies since favors the adhesion of NP complexes to the nasal mucosa. However, it has been shown that the NP movement is facilitated by low molecular weight PEG, as longer chains of PEG interact more with the mucosa slowing down the movement of the NPs [99]. Moreover, surface modifications with ligands, especially cell-penetrating peptides (CPPs), have been shown to be effective for enhancing NtB drug delivery. It was shown by Gartziandia et al. that only 0.7% of PLGA NPs were found in the nasal mucosa compared to 22% of chitosan-coated nanostructured lipid carriers (CS-NLC). When such NPs are coated with cell-penetrating peptides (CPPs) such as TAT and penetratin (Pen) these values increase up to 7 and 46% respectively [122]. Lecithins have also been often used to favor the adhesion and migration of NPs; however, these molecules have been found to be extremely immunogenic. Therefore, it is necessary to identify ligands that facilitate the permanence and migration of NPs through this pathway without being cytotoxic [111].

## 5. NtB Drug Delivery for the Treatment of Neurological Diseases

Diseases affecting the CNS are mainly represented by neurodegenerative diseases, characterized by a progressive and irreversible loss of neurons in specific regions of the brain, which can lead to cognitive deficits, dementia, motor alterations, behavioral and psychological disorders, and ultimately death. The most common neurodegenerative disorders are Parkinson’s disease (PD), in which the loss of neurons in the basal ganglia leads to abnormal movement control, and Alzheimer’s disease (AD), in which the degeneration of hippocampal and cortical neurons results in the loss of memory and cognitive abilities. However, there are a number of less common diseases, such as amyotrophic lateral sclerosis (ALS) and Huntington’s disease (HD), which also represent severe and potentially fatal conditions [123]. The socio-economic impact of these highly disabling pathologies undoubtedly represents one of the most serious health problems of the millennium. The major risk factor is represented by the age associated with the onset of these diseases. The chronic nature of these pathologies, together with the difficulties relating to their prevention, diagnosis, and therapy, make ever more urgent the need for finding truly effective treatments, rather than merely palliative cures [110]. To date, various strategies have been tested in order to deliver active drugs to the brain for the treatment of neurological disorders, including either intravenous or intranasal administration. In the latter case, however, the actual amount of drug reaching the brain has been shown to be less than 0.1%. In fact, the administration of therapeutic agents in their free form through the NtB route limits their absorption rate. To overcome this drawback, the bonding of therapeutics to specific carriers, which prevents drug degradation and facilitates their penetration through the nasal mucosa, has been carefully considered. In this regard, numerous studies have been conducted to demonstrate the high efficacy of NPs, in particular polymeric ones, to convey therapeutic molecules through the NtB route and some representative examples are listed in Table 3. In this regard, polymers such as chitosan and PLGA have been widely used, and numerous potential applications have been tested to evaluate the NtB treatment of neurodegenerative diseases. Bromocriptine-loaded chitosan NPs (BRC), for example, are effective in reducing symptoms in mouse models of PD. Similarly, this carrier has been used to deliver galantamine in AD mouse models, improving brain function and memory [124,125,126,127]. An interesting study conducted by Clementino et al. demonstrated the efficacy of simvastatin encapsulated in chitosan-lecithin NPs (SVT-LCN) for the treatment of neurodegenerative diseases, especially for AD where it favors a reduction in cholesterol levels and consequently the β-amyloid protein concentration. An in vitro release test was conducted by the dialysis bag diffusion method with which it was possible to demonstrate a faster release of molecule encapsulated in NPs than its suspension; in fact, after 8 h, about 40% of simvastatin was released from SVT-LCN against the 20% of simvastatin suspension. The fast release of simvastatin is also due to the degradation of the NPs that are attacked by the action of nasal secretions where lysozyme is mostly present. In fact, already after 1 h the outer shell of the NPs was degraded by enzymatic action, thus favoring the release of the drug [128]. PLGA NPs have also been extensively investigated. This is the case of levodopa, which, conveyed through this carrier, has provided a lasting recovery of motor function in the PD rat, or the bFGF which, administrated through PEG-PLGA composite NPs, has proved to determine an increase in cognitive abilities in AD models [129,130].

Neurodegenerative diseases also include lysosomal storage diseases (LSDs) caused by the absence or deficiency of specific enzymes of the lysosomal compartment that determine the accumulation of substrates in particular areas of the body, especially in the brain, resulting in a progressive loss of brain function and eventually death at a young age [3,48,49,123]. To date, for most LSDs, there are no definitive treatments other than palliative and supportive therapies, as the classic therapeutic approaches based on enzyme replacement therapy (ERT) and gene therapy (GT) usually fail to reach corrective levels of the deficient protein in the brain due to the insurmountable presence of the BBB. For this reason, the NtB approach could represent a promising therapeutic tool. As of today, there are few scattered studies regarding this novel and promising therapeutic approach to treating LSDs. For example, in 2018, Schuh et al. demonstrated that the nasal administration of nanoemulsions containing a plasmid encoding for the protein alpha-L-iduronidase (pIDUA) allowed corrective levels of the deficient enzyme to be reached in animal models of type I mucopolysaccharidosis (MPSI) [139].

The NtB approach is also closely studied for the treatment of glioblastoma multiforme (GBM), a malignant astrocytic tumor representing one of the most frequent oncological pathologies of the CNS. This type of neoplasia is characterized by extremely rapid growth and invasion of surrounding tissues. The therapeutic strategies currently used include surgery, radiotherapy, chemotherapy, and immunotherapy. However, traditional GBM treatments are ineffective for several reasons, such as the inability to remove the entire volume of tumor cells during surgery, the difficulty in reaching the tumor site due to the presence of the BBB, and the limitations of radiotherapy that is not able to eradicate radio-resistant GBM cells, especially cancer stem cells. Another peculiar feature of this type of tumor is the extensive vascularisation which, in addition to facilitating the expansion of the tumor and the migration of cells into the surrounding tissues, also determines the formation of a blood–tumor barrier (BTB) which makes it even more difficult the passage of chemotherapeutic drugs [140,141]. The use of nanotherapies in the treatment of GBM seems to bring significant advantages starting with an improvement in the targeting of cancer cells. It can be specifically exploited by a passive route named Enhanced Permeability and Retention effect (EPR effect), a mechanism present in the majority of human malignancies where particular conditions, such as an inflammatory state or hypoxia, make the endothelial lining of blood vessels more permeable, facilitating the molecule passage. However, although the use of NPs as a carrier of active molecules against GBM exploiting the EPR effect has given good results in mouse models, there are no clinical data in this regard because most of the clinical trials have been found to be fallacious [142,143]. There are other several routes of administration of NPs that can be considered for the treatment of GBM. As is the case with the other pathologies affecting the CNS, intracranial injection allows the drug to reach the tumor site directly, without the need to pass the BBB; however, this type of approach is extremely invasive and can produce dangerous side effects. Intranasal administration can be again considered a viable route of administration that allows the drug to reach the pathological site by overcoming the BBB and BTB in a less invasive way and with limited side effects for the patient. Furthermore, numerous studies have shown that by conveying active substances against GBM conjugated to NPs, much greater efficacy and concentration are obtained compared to the drug in its free form [140]. Several therapeutic agents are currently being studied for the treatment of GBM by exploiting the NtB route through the use of innovative formulations based on nanocarriers, which can also be modified on the surface in order to facilitate the release of drugs into specific cancer cells (Table 4). Most of the studies are currently only in a preclinical development phase, where the obtained data however show a better biodistribution and a better therapeutic effect of the anticancer compounds after intranasal administration [140].

## 6. Conclusions and Outlooks

The BBB represents the biggest obstacle for drugs intended to reach the brain and therefore for the treatment of all those pathologies involving the CNS, such as neurodegenerative and tumor diseases. Polymer NPs, due to their chemical and physical characteristics, lend themselves to being systems capable of by-passing the barrier represented by the BBB and thus transporting the therapeutic molecule into the brain. NPs are also able to protect the drug from biological and chemical degradation, increasing its bioavailability. The intracranial injection of nanoparticulate systems, however, is extremely invasive; furthermore, for disorders that require chronic treatment, such as those related to neurodegenerative diseases, non-invasive therapies would be desirable. For this reason, an alternative strategy is represented by the transport of active molecules through the NtB route. This pathway involves mainly the olfactory and trigeminal nerve systems starting in the brain and ending in the nasal cavity and represents the only portion of the CNS in close contact with the external environment, and consequently the most direct and non-invasive access route to the brain. To date, many studies have considered the administration of therapeutic drugs, peptides, and nucleic acids through intranasal administration for the treatment of neurodegenerative diseases and GBM, while its employment in the case of LSDs, another family of disorders affecting the CNS, is still in its early stages. In most of these studies, it has been shown that the NtB route allows high levels of drugs to be reached in the brain thanks to the use of nanocarriers with a diameter up to 200 nm in size, which can also undergo superficial modifications, such as using PEG or CPPs, to facilitate movement through the nasal route. These findings unlock the enormous potential of the intranasal delivery of therapeutics for brain targeting and also suggest that drugs can be effectively transported into the brain via the NtB route, thus avoiding systemic circulation. In addition, biopolymer NP carriers have proved to have a greater ability to transport the drug to the CNS and to increase its pharmacological activity when administered via the nose, as opposed to the simple administration of the drug in its free form. The combined advantages granted by both choosing the NtB route and resorting to biopolymer NPs, therefore, ensure an extremely efficient administration of drugs to the CNS. However, as of now, these studies have only been conducted in vitro or in murine models and thus require more clinical data on suitable animal models to evaluate the risks and benefits of the drug-loaded NPs and their efficacy in humans.

## Figures and Tables

**Table 1:** Advantages and disadvantages of the different strategies of the crossing of the blood–brain barrier (BBB).

Strategy	Advantages	Disadvantages	Ref.
Passive diffusion	Not require energy (ATP) consumption.	Only small lipophilic molecules (<500 Da) might diffuse.	[1,25]
Active diffusion	Transport a variety of molecules with structural diversity.	Require ATP consumption and restrict the passage of some therapeutic drugs.	[1,22]
Adsorption transcytosis	Molecules non-specifically bound to the membrane are internalized by endocytosis.	Slow and non-selective process.	[1,26]
Receptor-mediated transcytosis	Selective process specific for the largest molecules.	Slow process that requires the presence of specific receptors.	[1,27]

**Table 3:** Biopolymer NP carriers in the treatment of neurodegenerative diseases through the NtB route.

Pathology	Drug	NP Composition	NP Size	NP Synthesis Method	Biological Outcomes	Ref.
PD	BRC	CS	~160 nm	Ionic gelation	High-uptake of BRC-CS NPs via the NtB route and symptomatology reduction in PD mice.	[124]
PD	RH	CS	~170 nm	Ionic gelation	High accumulation of RH-CS NPs in the brain and higher mucoadhesion of RH-CS NPs than RH solution form in rats.	[131]
PD	Levodopa	CS	~100 nm	Ionic gelation	High accumulation and enhanced residence of levodopa-CS NPs in the brain of Wistar rats.	[132]
PD	Levodopa	PLGA	~250 nm	Emulsion/solvent evaporation	Intranasal levodopa-PLGA NPs provide a lasting motor function recovery with sustained effect in the 6-OHDA-induced PD rat model.	[129]
AD	Galantamine	CS	40–80 nm 180–190 nm	Ionic gelation	Intranasal galantamine-CS NPs improve the distribution of the drugs in different brain areas and ameliorate memory and brain functions in Wistar rats.	[126,127]
AD	Tarenflurbil	PLGA	~140 nm	Emulsification/solvent diffusion	Tarenflurbil-PLGA NPs improve drug bioavailability and brain targeting in SD rats.	[133]
AD	VIP	PEG-PLA	100–120 nm	Emulsion/solvent evaporation	VIP is a promising agent for the AD treatment. VIP-PLA NPs improve drug bioavailability in SD rats and KM mice.	[134]
AD	bFGF	PEG-PLGA	~110 nm	Emulsion/solvent evaporation	bFGF-PEG-PLGA NPs improve cognitive and memory ability in SD rats.	[130]
AD	NAP	PEG-co-PCL	70–90 nm	Emulsion/solvent evaporation	NAP-PEG-co-PCL improves cholinergic function and reduces neurodegeneration in SD rats and AD mice model.	[135]
AD	HupA	PLGA	~150 nm	Emulsion/solvent evaporation	HupA-PLGA NPs have a good sustained-release effect in KM mice.	[136]
HD	anti-HTT siRNA	CS	100–200 nm	Emulsion/solvent evaporation	Anti-HTT-siRNA-CS NPs determine a low expression of HTT mRNA in HD mice models.	[137]
HD	Cholesterol	g7-PLGA	~180 nm	Nanoprecipitation and simple emulsion	Cholesterol-(g7)-PLGA NPs enhance endogenous cholesterol biosynthesis, prevent cognitive decline, and ameliorate motor defects in HD mice.	[138]

Legend: Parkinson’s disease = PD; Alzheimer’s disease = AD; Huntington’s disease = HD; bromocriptine = BRC; ropinirole hydrochloride = RH; vasoactive intestinal peptide = VIP; neuroprotective peptide = NAP; huperzine A = HupA; huntingtin = HTT; heptapeptide = g7; chitosan = CS; poly lactic-co-glycolic acid = PLGA; polyethylene glycols = PEG; polycaprolactone = PCL; 6-hydroxydopamine = 6-OHDA; Sprague-Dawley = SD; Kunming = KM.

**Table 4:** BioPolymer NP carriers in the treatment of Glioblastoma (GBM).

Drug	NP Composition	NP Size	NP Synthesis Method	Biological Outcomes	Ref.
MLT	PCL	~170 nm	Nanoprecipitation	MLT-PCL-NPs exhibit a strong anticancer activity against U87MG cell line and an accumulation in the brain of Wistar rats.	[144]
DOX	RGD-PLGA	180–200 nm	Double emulsion method	DOX-RGD-PLGA NPs induce apoptosis and inhibition of brain tumor growth and in GBM rat model.	[145]
Bevacizumab monoclonal antibody	PLGA	~185 nm	Emulsion/solvent evaporation	Bevacizumab-PLGA NPs induce a reduction of tumor growth and show a higher anti-angiogenic effect in CD-1 mice.	[146]
anti-Gal-1 siRNA	CS	~170 nm	Ionic gelation	anti-Gal-1 siRNA-CS NPs reduce the expression of Gal-1 both in murine and human cells of GBM and in GBM mice.	[147]
CPt	PCL	~300 nm	Double emulsion/solvent evaporation	CPt-PCL NPs show high nasal absorption and high in vitro cytotoxicity in LN229 human GBM cells.	[148]
FTA	Lipid-PEG-PLGA	~160 nm	Emulsion/sonication method	Intranasal administration of FTA-lipid-PEG-PLGA-NP determines the reduction of 55% of the tumor area in GBM rats.	[149]

Legend: Glioblastoma = GBM; melatonin = MLT; doxorubicin = DOX; arginylglycylaspartic acid = RGD; galectin-1 = Gal-1; carboplatin = CPt; farnesylthiosalicylic acid = FTA; poly(ε-caprolactone) = PCL; poly lactic-co-glycolic acid = PLGA; chitosan = CS; polyethylene glycols = PEG.
